# Captivity alters behaviour but not seasonal brain size change in semi-naturally housed shrews

**DOI:** 10.1098/rsos.242138

**Published:** 2025-03-05

**Authors:** Cecilia Baldoni, Konstantinos Raptis, Marina Farantouri, Ivan Lenzi, Ka Sing Lim, Myles H. M. Menz, Marion Muturi, Marco Reisert, Maria Alejandra Bedoya Duque, William R. Thomas, Liliana M. Dávalos, John D. Nieland, Dominik von Elverfeldt, Dina K. N. Dechmann

**Affiliations:** ^1^ Department of Migration, Max Planck Institute of Animal Behavior, Radolfzell am Bodensee, Germany; ^2^ Department of Biology, University of Konstanz, Konstanz, Germany; ^3^ Computational and Analytical Sciences, Rothamsted Research, Harpenden AL5 2JQ, UK; ^4^ College of Science and Engineering, James Cook University, Townsville, Australia; ^5^ Department of Diagnostic and Interventional Radiology, Division of Medical Physics, University Medical Center Freiburg, Faculty of Medicine, University of Freiburg, Freiburg, Germany; ^6^ Department of Stereotactic and Functional Neurosurgery, Medical Center of Freiburg University, Medical Faculty of Freiburg University, Freiburg, Germany; ^7^ Department of Ecology and Evolution, Stony Brook University, Stony Brook, NY, USA; ^8^ Consortium for Inter-Disciplinary Environmental Research, Stony Brook University, Stony Brook, NY, USA; ^9^ Department of Health Science and Technology, Aalborg University, Aalborg, Denmark

**Keywords:** *Sorex araneus*, associative learning, chronic stress, motivation, magnetic resonance imaging, activity

## Abstract

Captivity, frequently used in animal research, can profoundly alter brain size, cognitive abilities and activity levels. Critically, persistent exposure to stressors in captive environments can lead to chronic stress and subsequently to a range of health issues. However, the direct implications of captivity on research outcomes have not been thoroughly investigated. We examined the effects of captivity on the common shrew, *Sorex araneus*, a species that exhibits a profound seasonal reversible change in brain and body size. We compared wild shrews during summer and winter to assess seasonal changes in brain size and behaviour and then contrasted these findings with shrews kept in captivity for six months. Using repeated *in vivo* magnitic resonance imaging, we determined that the extent of seasonal brain size change was not affected by the semi-natural captive conditions. However, captivity led to increased activity levels and reduced learning motivation in the shrews, indicative of chronic stress. These results suggest that even semi-natural conditions can significantly alter the outcome of studies and these effects need to be quantified before experimentation.

## 1. Introduction

Despite most animal research being conducted in captivity, the potential effects of the captive environment are rarely assessed. Captivity allows for controlled settings and standardized research approaches that are difficult to implement in complex natural environments. Yet, emerging evidence indicates captivity effects are profound and complex [1–3], making it challenging to accurately study natural processes, such as behaviours [4] but see [5]).

Captivity can lead to long- and short-term changes. Heritable changes can affect behaviour [6], brain size [7–9] and genome [10,11] in animals bred for laboratory research, domestication, food or fur production. These changes are often unintended byproducts of breeding in captivity, making many changes irreversible if animals return to the wild, resulting in lower survival [12–14] (but see [9]). In wild-caught animals, captivity can have short-term, potentially reversible impacts on many processes including behavioural and physiological adaptations. These changes stem directly from commonly used captive conditions, which expose the animals to chronic stressors such as intensive human interaction, confinement, diet, food-seeking behaviour or artificial lighting [15,16]. Such chronic stress leads to changes in hormone balance, immune responses [17–19] and gene expression [20–22], which can result in a variety of health issues, further affecting cognitive and physical abilities [23]. The cumulative impact of these factors can have additional behavioural effects such as altered activity levels in captive animals, ranging from lethargy to stereotypy [24]. Interestingly, the negative cognitive effects of captivity can be mitigated to some extent, particularly by enriched environments [25,26], suggesting that strategic modifications can reduce the profound effects of captivity on animal well-being.

It is essential to recognize the differences between captive and wild animals not only in behaviour but also in physiology and the underlying genetic mechanisms to understand the confounding effects of captivity on research [4,27]. The common shrew (*Sorex araneus*) serves as an ideal model for such studies, particularly due to its unique seasonal physiological adaptation known as Dehnel’s phenomenon [28,29].

The life cycle of the common shrew demonstrates the profound impacts of seasonal adaptations (see electronic supplementary material, figure S1). Born in summer, shrews grow rapidly, reaching their maximum brain size within just a few weeks (henceforth called summer juveniles). In anticipation of winter, they undergo the first stage of Dehnel’s phenomenon, a dramatic size reduction [28]. Dehnel’s phenomenon affects the whole body, bones and internal organs, to varying degrees across tissues and organs [30]. Even within the brain, different brain regions change size to different extents [31]. They reach a minimum brain, body and skull size as sexually immature subadults in February (winter subadults). The seasonal shrinkage is thought to help meet the energy demands of colder months and reduce food availability. As relative energy consumption remains constant, shrews lower absolute metabolic needs by reducing the body size and metabolically expensive organs, increasing the chances of survival during winter [32,33]. This enables shrews to remain active throughout the winter. In spring, shrews partially regrow their skull and brain and significantly increase in body mass—by 80−100%—to prepare for reproduction reaching a second size peak in June (summer adults). Simultaneously, shrews reach adulthood, become sexually mature, reproduce and die, their lifespan typically lasting about a year.

Shrinking in overall size and reducing the mass of energetically expensive organs such as the brain reduces energy consumption and food requirements, yet this reduction in brain size compromises function. Shrews freshly caught from the wild perform less effectively in a spatial navigation task in winter, the period of reduced brain size [34]. To work with captive shrews, it is important to be able to distinguish the effects of Dehnel’s phenomenon from those resulting from captivity. Previous work has identified significant changes in gene expression in this population after just two months of captivity, despite being kept in the semi-natural conditions described above [20], which involve gene families involved in depression, stress responses, and neurodegeneration, further highlighting the need to understand captivity effects.

A practical approach for studying this cycle and its effects is to capture the shrews as newly independent juveniles in summer, and then maintain them in captivity throughout their lifespan, which averages 13 months in the wild. This allows for repeated measurements of brain size and behavioural tests. Thus, it is important to determine how captivity influences these changes. When kept in semi-natural conditions in outdoor aviaries, skull size, a proxy for brain size, showed the same pattern of change in captive shrews as in their wild counterparts [35]. In contrast, when maintained indoors at a natural light cycle, but at a constant temperature, skull size steadily declined throughout their life. Those two studies showed how brain size change in shrews is flexible and influenced by environmental factors (see table 1 for an overview of known captive and seasonal effects on shrews).

**Table 1.** Overview of known seasonal and captivity effects on common shrew traits. The table summarizes skull size, brain size, cognition and activity findings. Seasonal effects refer to differences between summer and winter in wild populations, while captive effects refer to differences observed under semi-natural captive conditions compared to wild counterparts. Relevant literature references are provided in the final column.

topic	seasonal effects (wild shrews)	captivity effects	studies
skull size	*in vivo* and *ex vivo*: skull size shrinks seasonally from summer to winter	skull size changes in captive shrews exposed to natural temperature and light were identical to those in free-ranging shrews. No skull size change in shrews housed at constant temperature, but natural daylight patterns indoors	[28]; [36]; [35]
brain size	seasonal shrinkage and regrowth patterns previously observed *ex vivo*: current study *in vivo*	no prior studies; this study directly examines brain size under captive conditions *in vivo*	[31]; [37]; current study
cognition	seasonal effects in fresh caught wild shrews: summer juveniles show better spatial navigation skills than winter subadults	no prior studies; this study directly examines cognition under captive conditions	[34]; current study
activity	lower activity in winter compared to summer	no prior studies; current study examines the effect of captivity on activity	[38]; current study

To explore the effects of captivity, we compared the cognition, brain morphology and activity of young shrews in summer with subadults in winter. Here, we used two categories: shrews that had been in semi-natural captivity since summer and fresh-caught ones from the wild. Summer to winter is when the greatest change in shrew brain size occurs, and we hypothesized that even semi-natural captive conditions would alter the shrews’ behavioural and cognitive responses. First, we followed brain volume changes in shrews using *in vivo* magnetic resonance imaging (MRI), anticipating that the semi-natural conditions would not influence the natural seasonal reduction in brain size [35]. Then, we tested shrews in an associative learning task, considering how the cognitive impairment caused by Dehnel’s phenomenon might be influenced by captivity, which imposes environmental constraints absent in the wild, such as limited foraging opportunities and reduced sensory stimuli, as well as being exposed to a different diet. If these constraints significantly affected shrews kept in captivity, we anticipated a noticeable decline in cognitive functions. Conversely, if the semi-natural conditions sufficiently mimicked the wild environment, then the expected cognitive decline might be less pronounced or absent. Lastly, building on the finding that all shrews immediately begin using running wheels in captivity [38], we examined activity patterns between the three categories. Although wild shrews are less active in winter [39], we hypothesized that even semi-natural captive conditions would alter the shrews’ behavioural and cognitive responses. This could manifest as increased activity levels, similar to the restlessness observed in captive migratory birds [40], potentially indicative of chronic stress. Alternatively, if captivity does not significantly disrupt typical seasonal behaviours, we might observe activity patterns that resemble those of wild shrews, with reduced winter activity.

The impact of captivity is increasingly recognized as a factor that can significantly mask the effects of treatments or other experimental methods. Given the increasing interest in shrews for applied research, it is important to measure these effects at an early stage to establish protocols that account for the influence of captivity. These studies are crucial for understanding how captivity not only affects animal physiology and behaviour but also influences the outcomes of experimental research.

## 2. Methods

All handling and sampling methods were approved by the Regierungspräsidium Freiburg, Baden-Württemberg, Germany (35-9185.81/G-19/80 and 35-9185.81/G-22/082). Common shrew individuals were caught from the wild in Möggingen, Germany (47°46′04.70″ N, 8°59′47.11″ E), near the Max Planck Institute of Animal Behaviour. We caught 13 shrews with live wooden traps (PPUH A. Marcinkiewicz, Rajgród, Poland) in August 2021 when their brain size was close to its maximum (from now on referred to as summer wild) and kept them in captivity until February 2022 (six months) when brain size was at a minimum (winter captive). As is typical for this species, mortality occurred in autumn, and by February 2022, our captive group had been reduced to seven individuals. Due to the rapid physiological changes inherent to Dehnel’s phenomenon, it was not possible to conduct both behavioural and imaging experiments within the narrow time frame required to capture the peak of minimum brain size. Consequently, we caught nine additional shrews (winter wild) by February 2023, to be compared with our captive winter subadults. The mean temperature in both years was similar: 2.82 in 2022 and 2.84 in 2023 [41]. We did not determine the sex of the shrews in our experiments. Shrews typically reach sexual maturity in spring (April–May), but we tested them in summer and winter. Previous research indicated no significant differences between males and females regarding brain shrinkage and cognitive abilities [34] during the non-reproductive seasons. All shrews were housed individually in a double-cage system. The primary cage contained a thin layer of soil, a running wheel, food and water dishes and a small flowerpot filled with hay that served as a nest and shelter. This cage was connected via a plastic tube to a secondary cage, which served as a nesting area, filled with multiple layers of soil and hay (see electronic supplementary material, figure S2). Shrews were fed daily with a specially prepared raw meat mixture [42] supplemented with 2 g of mealworms (*Tenebrio monitor* larvae). The daily food amount was adjusted monthly based on the shrews’ average body weight (e.g. an 8 g shrew would receive 8 g of food) [43]. Water was provided ad libitum. All shrews were housed in an outdoor aviary, with each cage placed on separate shelves to prevent individuals from seeing one another. In the aviary, shrews were exposed to natural light, temperature and humidity, which we consider to be semi-natural captive conditions. We carried out all MRI scans and cognitive tests and collected running wheel data within four weeks each season to coincide with brain size peaks—its maximum in summer and minimum in winter. This method helped to minimize the confounding effects of brain size fluctuations by capturing data at these narrow time peaks when changes are most pronounced.

### 2.1. Brain volumes

For summer wild, winter captive and winter wild, we scanned 11, 7 and 9 shrews, respectively. MRI scans of the brain were performed at the Universitätsklinikum Freiburg, Germany, using a BioSpec70/20 system (Bruker Biospin, Ettlingen, Germany) equipped with a BGA12S gradient insert, and with a cryogenically cooled 2-channel Tx/Rx mouse head surface coil. We transported individual shrews to Freiburg inside their home cages and returned them to their holding facilities within 10 hours. We induced anaesthesia in a knockout chamber utilizing 3.5–4% sevoflurane in O_2_ with a gas flow rate of 1.9 l min^−1^. We then transferred them to an animal bed with a custom three-dimensional-printed nose holder, ensuring a continuous supply of 2.5–3% sevoflurane (gas flow rate 1.2 l min^−1^ with one-third O_2_ and two-thirds air). We maintained a constant body temperature with a warm water flow tube under the shrew and monitored the breathing rate with a respiratory pad beneath the shrew’s abdomen. We recorded overall brain anatomy, which took 20 min or less. Following measurements, we returned the shrews to the knockout chamber, where they received pure oxygen until awakening, a process that typically takes only a few seconds. We then returned the shrews to their home cage with access to water and food and continuously observed them until the effects of anaesthesia had completely worn off.

### 2.2. Magnetic resonance imaging data acquisition and validation

Following an oblique pilot scan, brain anatomy was recorded using a T_2_ weighted Turbo RARE sequence with axial orientation, and the following parameters: TE/TR = 40 ms/5075 ms, 2 averages, RARE-factor = 8, 40 slices, FOV = 16 mm × 12 mm, slice thickness of 0.3 mm, matrix size 200 × 150, isotropic in-plane resolution 80 µm, bandwidth 35 kHz, and a total acquisition time of 3 min and 2 s. To quantify brain volume, we followed the traditional approach of voxel-based morphology [44]. Therefore, we first constructed a shrew brain template based on a group of animals. The template is then used as a reference to coregister individual animals in a deformable manner into template space, and the determinant of the Jacobian matrix of the warp is used to quantify the brain volume locally. A high-resolution post-mortem scan of a shrew served as a starting point for the template space [45]. Based on brain-masked and bias field-corrected T2w scans the ANTs toolbox was used for co-registration into the template space. After the coregistration of 50 animals, the mean T2w contrast in template space is computed, and the mean is again used as a reference contrast in template space to coregister the same 50 animals, which results in a final template image. The ANTs toolbox [46] was used for all registration steps and mutual information as a similarity measure.

### 2.3. Associative learning task

For the associative learning task, we tested 11 summer wild, 7 winter captive and 8 winter wild individuals. We evaluated the ability of shrews to associate an odour cue with a reward (associative learning task) in a Y-maze (graphic depiction in electronic supplementary material, figure S3). For this, we selected five artificial food flavours as cues (‘odours’, i.e. vanilla, lemon, orange, almond, sugar cane). Before running the experiment, we ensured that the five odours were neither strongly preferred nor actively avoided by running six exposure trials (details in electronic supplementary material). All five odours were randomly assigned as correct or incorrect cues for each individual, except orange and lemon, which we never presented simultaneously because their odours are isomers of the same molecule.

We conducted the main experiment on the day after the exposure trials as follows. We food-deprived the shrews for 2 h, slightly longer than their natural feeding interval. We transported the shrews to the experimental room in their homecage, which was then connected to the Y-maze apparatus with a flexible tube. Shrews were then able to decide freely when to enter the Y-maze. Each arm of the Y-maze led to a box with a one-way door forcing the shrew to make an irreversible decision. These boxes contained one of two odour cues, applied to a sponge in a placeholder glued to the top of the box. Each box also contained either the scent of mealworms (unrewarded box) or a frozen mealworm (rewarded box), which was not visible from outside the box. As shrews do not exhibit high levels of food motivation in captivity, we additionally connected the rewarded box to the home cage allowing the shrew to return to the safety of the homecage as an additional incentive (adapted from [47]). An equally long dead-end tube was connected to the unrewarded box. After each shrew made a decision and entered a box, the choice was recorded as either correct or incorrect. A correct choice was defined as entering the rewarded box containing the mealworm. Following a correct choice, the shrew could return to the homecage through a connecting tube. If the shrew entered the unrewarded box, it was forced to remain in the dead-end tube for a brief period (approx. 10 min) before being returned to the homecage by the experimenter. After each trial, we cleaned the Y-maze with alcohol to remove scent marks, replaced the boxes and positioned them on their designated sides for the next trial. Each shrew performed 10 consecutive trials and was then returned to the aviary in the homecage.

### 2.4. Running wheel activity

We designed a running wheel system using a similar approach applied to construct computerized tethered flight mills for studying insect behaviour, to investigate activity patterns in the shrews [48]. We attached a black-and-white striped laminated paper ring (16 cm diameter, 1 cm height) to the outside rim of a commercially available mouse running wheel (16 cm diameter). A light sensor was fixed to an L-shaped wire arm attached to the wheel frame. The light sensor was positioned so that the black-and-white ring could pass through the sensor to detect rotations of the wheel, with the circumference of one rotation measuring 50 cm. The sensor was connected to a microcontroller board with eight channels, allowing up to eight running wheels to be operated simultaneously. Data were captured using custom software run on Microsoft Windows 11. Data recorded were distance moved (m), time spent running (s) and running speed (m s^−1^). Data for each shrew were extracted via a MATLAB script (The MathWorks Inc. 2022). Shrews begin using the running wheel within the first hour of being placed in captivity. Shrews had constant access to the wheels and could choose when to run. All bouts of running were recorded in August–September 2021 for wild summer shrews and in February–March 2022 and 2023 for the wild and captive winter shrews. Data were collected in all three cases for four weeks. We collected a total of 19 737 running bouts. To reduce the likelihood that recordings may have been due to movement from other influences such as the shrew brushing against the wheel, only bouts that were at least 0.3 m were included in the analyses. This corresponds to the wheel moving at least half a turn, or roughly four body-lengths of a shrew.

### 2.5. Statistical analyses

All analyses were performed in R version 4.3.1 (R Core Team, 2015). All models were developed using the brms package for Bayesian modelling. All our models were stable with large effective sample sizes (Bulk ESS and Tail ESS over 1000 for all estimates) and R-hat values smaller than 1.01. Pareto k estimates were below 0.5 for all models. We used prior and posterior predictive check functions to visually assess priors and model fit. For all models, we included a random intercept for each individual (ID) to account for repeated measures of some individuals (between summer wild and winter captive). All models and data can be found in the Edmond repository (see Data Availability statement).

**Brain volumes**: We conducted a Bayesian mixed effect analysis to determine differences in brain volume between categories (summer wild, winter captive and winter wild). We compared the effect of categories on brain volume.

**Associative learning task**: We compared the associative learning performance between wild summer juveniles, wild winter subadults and captive winter subadults. The model was based on a Bernoulli distribution for binary data (success = 1, failure = 0). We compared the effect of trial number in each category (summer wild, winter captive, winter wild) on success with generalized additive models (GAMs). We fitted the model with mild regularizing priors.

To ensure the accurate interpretation of our findings regarding individual associative learning abilities, we conducted additional analyses to rule out alternative strategies, such as reinforcement learning, side preference or random choice (see electronic supplementary material). Finally, we investigated the effect of category on latency (described as the time from the start of the experiment until the shrew entered the Y-maze) by implementing a hurdle model (log-normal) to account for the large proportion of instances where latency was zero.

**Running wheel activity**: We used a Bayesian hierarchical model to analyse whether the logarithm of the distance run was influenced by a cyclic spline by hour of the day for each category (summer wild, winter wild or winter captive) and average speed. Additionally, the model allowed the scale parameter (sigma) to vary by group. For the cyclic spline, ‘hour’ was defined as a 24 h period, capturing the cyclical nature of time throughout the day.

Finally, to test whether success in the cognitive task was influenced by brain volume or activity levels, we ran two additional models incorporating all three variables (see electronic supplementary material).

## 3. Results

### 3.1. Brain volumes

**Figure 1.**
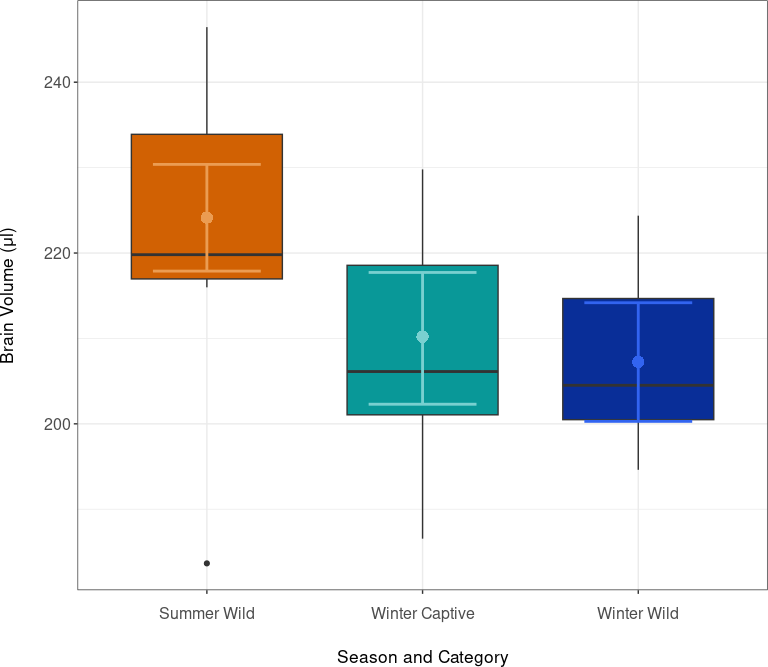
Brain volume comparison across the seasonal categories. The boxplots display brain volumes in microlitres. Points with error bars represent the posterior means and 95% credible intervals (CIs) derived from the Bayesian model.

When we compared wild summer juveniles with wild and captive winter subadults, brain volume changed as predicted based on Dehnel’s phenomenon. The brain volume in summer was 224.15 μl, which decreased to 210.19 μl in captive winter individuals and 207.25 μl in wild winter individuals (table 2). This is a reduction of approximately 7.54% from summer to winter captive and approximately 6.22% from summer to winter wild (figure 1). Similarly, there was no difference in the changes of individual brain regions (see electrnic supplementary material for detailed models). To address the smaller sample sizes in winter, we calculated contrasts between winter captive and winter wild brain sizes. The analysis revealed a mean difference in brain volume between the categories winter wild and winter captive of −2.94 units (95% credible interval: −13.22 to 7.22), indicating no statistically significant difference (see electronic supplementary material).

**Table 2.** Result summary from Bayesian model estimates with lower and upper 95% credible interval on brain volume, associative learning performance and running wheel activity in common shrews of three categories: summer wild, winter wild and winter captive.

brain volume	category	volume estimate (μl)	l−95% CI	u−95% CI
	*summer wild*	224.15	217.88	230.38
	*winter wild*	207.25	200.29	214.17
	*winter captive*	210.19	202.29	217.72
**associative learning**	**category**	**success (probability)**	**l−95% CI**	**u−95% CI**
	*summer wild*	0.642	0.598	0.681
	*winter wild*	0.629	0.575	0.679
	*winter captive*	0.608	0.563	0.653
**running wheel speed**	**category**	**estimate (m s^−1^)**	**l−95% CI**	**u−95% CI**
	*summer wild*	0.435	0.421	0.449
	*winter wild*	0.071	0.069	0.073
	*winter captive*	0.496	0.488	0.503

### 3.2. Associative learning task

**Figure 2.**
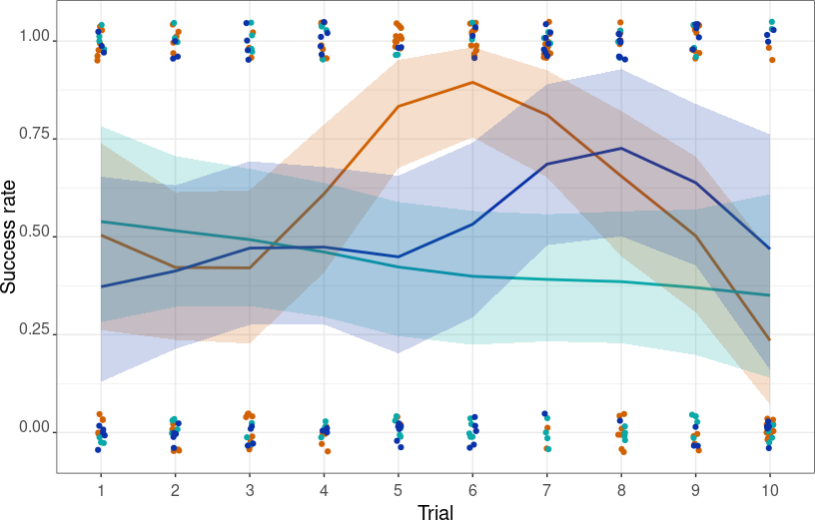
Success rate by category in the associative learning task. The solid lines show the estimated success rate across trials for three categories: summer wild (orange), winter captive (light blue) and winter wild (dark blue). Shaded areas indicate the 95% credible intervals (CIs). Jittered points are the observed values recorded in each trial for the respective categories.

The success rate between wild summer juveniles, wild winter subadults and captive winter subadults differed. In summer, the predicted probability of success was 0.642 (95% CI: 0.598 to 0.681). For animals in the winter captive category, the predicted probability of success was slightly lower at 0.608 (95% CI: 0.563 to 0.653). Similarly, the predicted probability of success for animals in the winter wild category was 0.629 (95% CI: 0.575 to 0.679). The seasonal trajectory of success rates over trial numbers also differed. In summer, the success rate increased from 0.504 in trial 1 to a peak of 0.894 in trial 6, then declined to 0.235 by the last trial. Winter wild individuals mirrored this pattern but peaked slightly later, in trial 8 (estimate trial 1 = 0.376, estimate trial 8 = 0.725) (figure 2; electronic supplementary material, table S3). Winter captives achieved their best performance during the first trial, and after that, performance declined with estimates 0.386 (95% CI = 0.230, 0.571) and 0.348 (95% CI = 0.136, 0.609) at trials 8 and 10, respectively.

Latency to enter the setup was higher in summer (estimate = 8.68 min, 95% CI = 5.77 to 12.28), compared to winter captive (estimate = 6.09 min, 95% CI = 3.70 to 9.39) and winter wild (estimate = 7.55 min, 95% CI = 4.41 to 12.17). The probability of zero latency was lowest in the winter captive category (0.015, 95% CI = 0.0009 to 0.056), followed by the summer wild category (0.11, 95% CI = 0.065 to 0.173). The highest probability of zero latency was observed in the winter wild category (0.39, 95% CI = 0.284 to 0.504), meaning that winter wild individuals entered the arena within a minute in 39% of the trials.

Regarding the model testing the effects of brain volume and activity on success, neither brain size nor activity explained variation in cognitive task performance (see electronic supplementary material).

### 3.3. Running wheel activity

**Figure 3.**
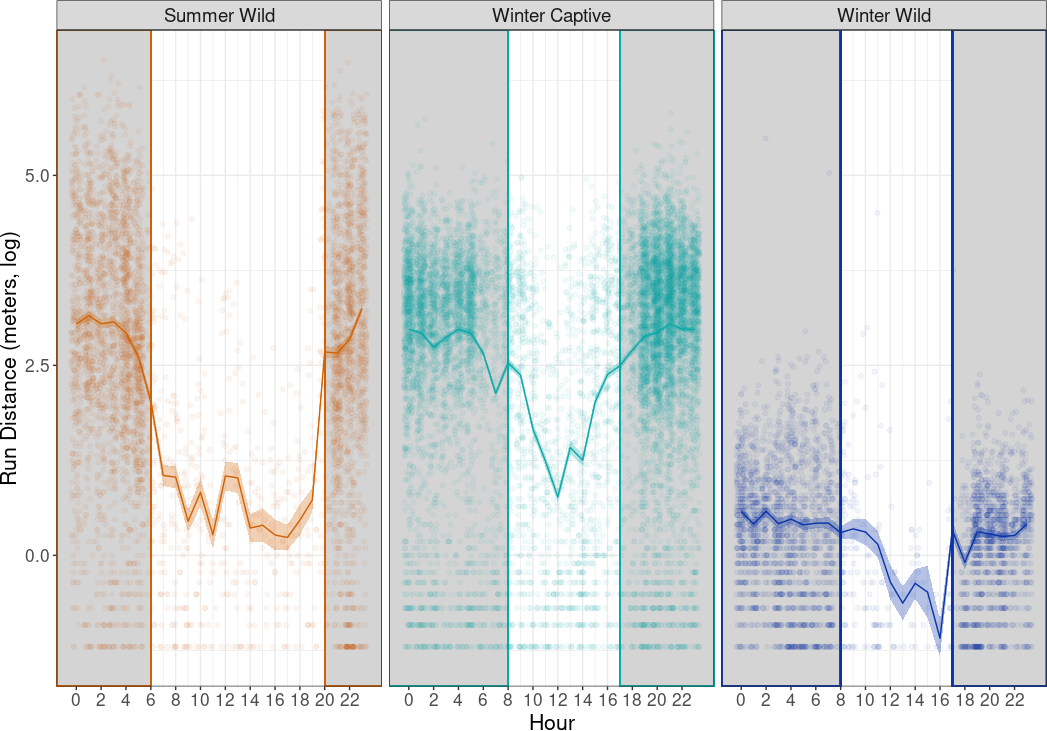
Running distances (metres, log-transformed) across the day for summer wild (orange line), winter captive (light-blue) and winter wild (dark-blue) shrews, with corresponding 95% CIs. Individual observations are shown as jittered points for each hour and category. Vertical grey lines separate day (white) from night (grey shaded) periods. In the summer wild category, sunrise and sunset occur at 06.00 and 20.00, respectively, while in winter captive and winter wild, sunrise and sunset were at 08.00 and 17.00, respectively.

All numerical results are presented in their original units (metres) in table 2, while figure 3 illustrates the data on a log-transformed scale to enhance the visualization of differences across groups. In summer wild individuals, the minimum run distance was observed at 17.00 with a value of 1.27 m (95% CI: 1.08, 1.49), and the maximum was observed at 23.00 with a value of 25.7 m (95% CI: 24.2, 27.3). For key hours corresponding to sunrise (06.00) and sunset (20.00), the estimated run distances were 7.62 m (95% CI: 6.97, 8.31) and 14.5 m (95% CI: 13.4, 15.7), respectively. In the winter captive category, the minimum run distance was observed at 12.00 with a value of 2.16 m (95% CI: 1.99, 2.34), and the maximum was observed at 21.00 with a value of 21.0 m (95% CI: 20.3, 21.7). At sunrise (08.00) and sunset (17.00), the estimated run distances were 12.6 m (95% CI: 11.9, 13.3) and 12.1 m (95% CI: 11.5, 12.8), respectively. For the winter wild category, the minimum run distance was observed at 16.00 with a value of 0.336 m (95% CI: 0.264, 0.425), and the maximum was observed at 2.00 with a value of 1.79 m (95% CI: 1.67, 1.91). At sunrise (08.00) and sunset (17.00), the estimated run distances were 1.35 m (95% CI: 1.24, 1.47) and 1.39 m (95% CI: 1.24, 1.56), respectively (see electronic supplementary material, table S5, for hour specific run distances). The winter wild group exhibited the lowest run distances overall, with less pronounced fluctuations throughout the day compared to the other categories. For all categories, run distance for daytime was generally lower compared to nighttime.

Regarding average speed, summer wild individuals exhibited an average speed estimate of 0.435 m s^−1^ (95% CI: 0.421, 0.449). In comparison, the winter wild category had a lower average speed estimate of 0.071 m s^−1^ (95% CI: 0.069, 0.073). The winter captive category showed the highest average speed among the groups, with an estimate of 0.496 m s^−1^ (95% CI: 0.448, 0.503).

## 4. Discussion

We assessed seasonal and captivity effects on common shrews, to tease apart their influence on patterns in brain volume, cognitive performance and activity. Brain volume decreased significantly from summer to winter, with no notable difference between wild and captive individuals. Cognitive performance varied across seasons and captivity status. Activity patterns demonstrated pronounced differences, with summer wild and winter captive individuals being more active overall, while winter wild individuals showed the lowest levels of activity and less variation throughout the day.

Despite the attenuated environment of captivity, there were no significant differences in overall brain volume in winter between wild common shrews and those held in semi-natural captive conditions for six months (figure 1, table 2). This aligns with skull measurements by Lázaro *et al.* [35] suggesting that Dehnel’s phenomenon remains unaffected under the same captive conditions. However, the range of the 95% credible interval (−13.22 to 7.22 ml; electronic supplementary material, table S1) suggests that the data are compatible with both a significant reduction and a modest increase in brain volume due to captivity. For instance, the interval includes effect sizes that could represent either half the brain size reduction observed in wild individuals or nearly twice the reduction. Therefore, while our results do not show a statistically significant difference, the potential biological impact of captivity on brain volume remains uncertain. While the reduction is generally consistent across regions, there is a slight variance in the degree of the reduction among brain regions with the detailed neuroimaging presented here (see electronic supplementary material). Although these differences are not statistically significant, they suggest some brain regions respond differently to captivity. Overall brain volume reduction from summer to winter was approximately 5.63% in shrews caught in summer and kept until winter, and 6.78% between the same summer shrews and freshly caught individuals in winter. Size reduction was less pronounced than the 10–26% decrease in braincase height from the same population in recent years [34]. While MRI directly measures the brain’s soft tissue, X-ray and braincase height measurements are based on the external dimensions of the skull, likely explaining the much lower estimates of size plasticity noted here. Non-exclusively the increasingly warm winters could be affecting the changes, which are known to be flexible [35].

Summer wild shrews outperformed their wild winter counterparts in the associative learning task (figure 2). This matches our expectations based on the reduced volume of the brain and specific brain regions associated with cognitive processing in winter. Lázaro *et al.* [35] reported a similar seasonal pattern in cognitive abilities in a spatial navigation task. However, the winter captive group performed even more poorly than the wild winter shrews. This could be due to additional cognitive impairment caused by captivity or lower motivation to participate in the task similar to the loss of interest in reward and pleasure in chronically stressed mice [49]. Winter captives also took longer to enter the arena for the cognitive test (see electronic supplementary material). Forty percent of the wild winter shrews entered the arena within a minute, but none of the winter captives did so. Poor performance and increased latency indicate that captivity affected the shrews’ overall behaviour, lowering their interest to participate in tasks they had previously engaged with. Various captivity-related factors, such as stress, altered environmental dynamics or a lack of natural stimuli, may explain the reduced motivation observed in the winter captive group. Differences between wild and captive winter shrews might be enhanced by cognitive impairment. Although even slight differences in specific brain regions may influence behaviour, the consistent brain size change observed in wild and captive categories links behavioural differences observed here to captivity instead. In a dynamic environment teeming with challenges, despite similar brain volume, wild shrews may have developed stronger cognitive skills, while captive shrews lack this environmental stimulation resulting in altered behaviour.

Continuous access to food in captivity may also diminish food-related motivation, which could explain the observed lower success rates and increased latencies in the behavioural test among winter captives. Conversely, while wild individuals might initially exhibit higher food motivation, the decline in correct choices in later trials could be attributed to the monotony of the same reward. Before the associative task with summer wild juveniles, we observed food preference, presenting them with two food items in different combinations (mealwork, earthworm, raw meat, low-sodium dog food). Shrews did not show a clear preference for any of the food types. Despite the inconclusive result from the food preference test, the consistent use of the same reward might have led to the observed decline in correct choices. Providing a different set of food rewards in later trials would account for this in the future (i.e. ‘variety effect’ [50,51]).

If the stress of captivity and not impaired cognition caused cognitive test performance declines, changes in overall activity should also be evident. Supporting a captivity effect, the captive winter group increased running compared to their wild counterparts. To conserve energy and resources in the wild, common shrews naturally decrease their activity in winter when food is more scarce and conditions are harsh [39]. This was also the pattern in both wild categories, with summer shrews running longer distances, mostly during the night, than winter shrews. In line with this result, winter captives also showed the highest average running speeds. The conditions of captivity resulted in higher running speeds compared to the wild conditions in both summer and winter. Several factors may contribute to this. Chronic stress or anxiety in captive animals is often tied to their housing conditions (e.g. long-term confinement, limited space and constant human presence) [16], which can disrupt the animals’ normal behaviour and physiological state. This matches the lack of motivation our captive shrews showed in the associative learning task. In some cases, anxiety or chronic stress can result in stereotypic activity, e.g. pacing, over-grooming, or, as in our shrews, excessive use of running wheels [52]. Hyperlocomotion has been identified as a confounding response in chronically stressed laboratory mice [53], and in captive wild animals this could be an attempt to cope with the psychological impact of confinement or to self-stimulate in an environment that lacks natural stimuli. Both heightened physical activity and a decrease in motivation for a reward can be adaptive responses to chronic stress caused by the artificial conditions of captivity.

Another non-exclusive explanation is the stress caused by the inability to perform natural foraging behaviour. In their natural habitat, common shrews forage frequently because of an extremely high metabolism that requires them to eat every few hours to survive [54,55]. In captivity, food is delivered every 24 h at the same time and location. As a result, the energy that wild shrews would typically expend on foraging may be redirected in captivity, leading to excessive running in winter.

The detailed mechanisms underlying the changes in activity and cognition we found remain unknown. However, our observations match recent findings from a parallel study comparing wild shrews with shrews kept in captivity for two months, which documented significant changes in gene expression related to the stress response [20]. Specifically, captive shrews show downregulation of FKBP5 across three brain regions (−1.56 LFC in the cortex, −1.38 LFC in the hippocampus, −1.70 in the olfactory bulb), whose encoded protein is found in a negative feedback loop with the glucocorticoid receptors which has been shown to induce changes in mood, behaviour and other downstream physiological stress responses. Human individuals with post-traumatic stress disorder consistently have decreased levels of FKBP5, which dampens the cortisol response [56]. Coupled with increased running and lower cognitive performance or motivation, the downregulation of FKBP5 in captive shrews suggests a complex adaptive response to the stress of captivity. Downregulation of FKBP5 might be an attempt to enhance stress resilience by improving glucocorticoid receptor sensitivity to cortisol [57]. This improved sensitivity allows the body to regulate the stress response, facilitating the shutdown of the stress pathway once the stressor is removed. While stress resilience may enhance the ability to cope with stress, it can also lead to diminished cognitive performance and motivation due to the prioritization of coping mechanisms over other functions. In a chronically stressed state, the organism allocates resources towards survival and stress management, which can impair functions like learning, memory and motivation [58]. This shift in resource allocation can result in a tradeoff, in which increased resilience to stress comes at the expense of cognitive capabilities and engagement in tasks requiring higher cognitive effort.

Investigating epigenetic and expression changes in genes in this and other pathways known to be involved in chronic stress, such as the serotonin or dopamine systems in the brain or regions of the brain will help us better understand observed differences between wild and captive shrews and interpret results, especially those gathered for applied research [59–61].

In conclusion, semi-natural captive conditions did not significantly disrupt the natural pattern of seasonal brain size variation in common shrews over six months. Our study showed the effect of semi-natural captivity on common shrews, but it is important to use these findings carefully when applying them to other species. Shrews are characterized by their high metabolic rates and insectivorous diet, which necessitate continuous activity throughout the year as they do not hibernate or enter torpor. These traits may limit the applicability of our results to species with different ecological roles or environmental adaptations. Furthermore, the semi-natural conditions of our study, designed to replicate the shrews' natural daylight and temperature, may not directly compare with those used in conventional laboratory studies involving other species. Such differences in captive environments could influence the typical stress responses associated with captivity, potentially affecting the generalizability of our results. However, our research has shown the profound impact of captivity on the behaviour and possibly cognition of common shrews that we attribute to the complex interplay between environmental stimulation and stress responses. There is surprisingly little information on such captive effects and how they have been dealt with in other species, but quantifying these behavioural changes is crucial for both the welfare of animals in captivity and to interpret the results of research studies that use captive animals, as these conditions can influence the results of experiments.

Moving forward, it would be valuable to conduct comparative studies involving species with varying ecological and physiological traits to gain a more comprehensive understanding of how captivity affects behaviour across different taxa. By doing so, we can contribute to a broader understanding of the implications of captivity on animal cognition and behaviour while accounting for the diverse ecological and physiological factors that shape these responses.

## Data Availability

All data and code for the analysis and plots of this paper can be found in the Edmond repository [62]. Electronic supplementary material is available online [63].

## References

[1] Clubb R , Mason G . 2003 Captivity effects on wide-ranging carnivores. Nature **425** , 473–474. (10.1038/425473a) 14523435

[2] Marino L , Rose NA , Visser IN , Rally H , Ferdowsian H , Slootsky V . 2020 The harmful effects of captivity and chronic stress on the well-being of orcas (Orcinus orca). J. Vet. Behav. **35** , 69–82. (10.1016/j.jveb.2019.05.005)

[3] Schmidt E , Mykytczuk N , Schulte-Hostedde AI . 2019 Effects of the captive and wild environment on diversity of the gut microbiome of deer mice (Peromyscus maniculatus). ISME J. **13** , 1293–1305. (10.1038/s41396-019-0345-8) 30664674 PMC6474230

[4] Rees PA . 2015 Studying captive animals: a workbook of methods in behaviour, welfare and ecology. Hoboken, NJ: John Wiley & Sons.

[5] Cauchoix M , Hermer E , Chaine AS , Morand-Ferron J . 2017 Cognition in the field: comparison of reversal learning performance in captive and wild passerines. Sci. Rep. **7** , 12945. (10.1038/s41598-017-13179-5) 29021558 PMC5636806

[6] Crates R , Stojanovic D , Heinsohn R . 2023 The phenotypic costs of captivity. Biol. Rev. **98** , 434–449. (10.1111/brv.12913) 36341701

[7] Burns JG , Saravanan A , Helen Rodd F . 2009 Rearing environment affects the brain size of guppies: lab‐reared guppies have smaller brains than wild‐caught guppies. Ethology **115** , 122–133. (10.1111/j.1439-0310.2008.01585.x)

[8] Lesch R , Kitchener AC , Hantke G , Kotrschal K , Fitch WT . 2022 Cranial volume and palate length of cats, Felis spp., under domestication, hybridization and in wild populations. R. Soc. Open Sci. **9** , 210477. (10.1098/rsos.210477) 35116138 PMC8790375

[9] Pohle AK , Zalewski A , Muturi M , Dullin C , Farková L , Keicher L , Dechmann DKN . 2023 Domestication effect of reduced brain size is reverted when mink become feral. R. Soc. Open Sci. **10** , 230463. (10.1098/rsos.230463) 37416828 PMC10320332

[10] Araki H , Cooper B , Blouin MS . 2007 Genetic effects of captive breeding cause a rapid, cumulative fitness decline in the wild. Science **318** , 100–103. (10.1126/science.1145621) 17916734

[11] Larson G , Burger J . 2013 A population genetics view of animal domestication. Trends Genet. **29** , 197–205. (10.1016/j.tig.2013.01.003) 23415592

[12] Frankham R . 2008 Genetic adaptation to captivity in species conservation programs. Mol. Ecol. **17** , 325–333. (10.1111/j.1365-294x.2007.03399.x) 18173504

[13] Jule KR , Leaver LA , Lea SEG . 2008 The effects of captive experience on reintroduction survival in carnivores: a review and analysis. Biol. Conserv. **141** , 355–363. (10.1016/j.biocon.2007.11.007)

[14] Zeder MA . 2012 Pathways to animal domestication. In Biodiversity in agriculture (eds P Gepts , TR Famula , RL Bettinger , SB Brush , AB Damania , PE McGuire , CO Qualset ), pp. 227–259. Cambridge, UK: Cambridge University Press. (10.1017/CBO9781139019514.013)

[15] Fischer CP , Wright-Lichter J , Romero LM . 2018 Chronic stress and the introduction to captivity: how wild house sparrows (Passer domesticus) adjust to laboratory conditions. Gen. Comp. Endocrinol. **259** , 85–92. (10.1016/j.ygcen.2017.11.007) 29170021

[16] Morgan KN , Tromborg CT . 2007 Sources of stress in captivity. Appl. Anim. Behav. Sci. **102** , 2006. (10.1016/j.applanim.2006.05.032)

[17] Dickens MJ , Delehanty DJ , Romero LM . 2009 Stress and translocation: alterations in the stress physiology of translocated birds. Proc. R. Soc. B **276** , 2051–2056. (10.1098/rspb.2008.1778) PMC2677253 19324794

[18] Love AC , Lovern MB , DuRant SE . 2017 Captivity influences immune responses, stress endocrinology, and organ size in house sparrows (Passer domesticus). Gen. Comp. Endocrinol. **252** , 18–26. (10.1016/j.ygcen.2017.07.014) 28733227

[19] Seeber PA , Morrison T , Ortega A , East ML , Greenwood AD , Czirják GÁ . 2020 Immune differences in captive and free-ranging zebras (Equus zebra and E. quagga). Mamm. Biol. **100** , 155–164. (10.1007/s42991-020-00006-0)

[20] Bedoya Duque MA , Thomas WR , Dechmann DKN , Nieland J , Baldoni C , von Elverfeldt D , Muturi M , Corthals AP , Dávalos LM . 2025 Gene expression comparisons between captive and wild shrew brains reveal captivity effects. Biol. Lett. **21** , 20240478. (10.1098/rsbl.2024.0478) 39772919 PMC11706642

[21] DuRant S , Love AC , Belin B , Tamayo-Sanchez D , Santos Pacheco M , Dickens MJ , Calisi RM . 2020 Captivity alters neuroendocrine regulators of stress and reproduction in the hypothalamus in response to acute stress. Gen. Comp. Endocrinol. **295** , 113519. (10.1016/j.ygcen.2020.113519) 32470473

[22] Morales MH , Sánchez EJ . 1996 Changes in vitellogenin expression during captivity-induced stress in a tropical anole. Gen. Comp. Endocrinol. **103** , 209–219. (10.1006/gcen.1996.0112) 8812375

[23] Li S , Wang C , Wang W , Dong H , Hou P , Tang Y . 2008 Chronic mild stress impairs cognition in mice: from brain homeostasis to behavior. Life Sci. **82** , 934–942. (10.1016/j.lfs.2008.02.010) 18402983

[24] Resende L de S , Neto GL e , Carvalho PGD , Landau-Remy G , Ramos-Júnior V de A , Andriolo A , Genaro G . 2014 Time budget and activity patterns of oncilla cats (Leopardus tigrinus) in captivity. J. Appl. Anim. Welf. Sci. **17** , 73–81. (10.1080/10888705.2014.856253) 24484312

[25] Salvanes AGV , Moberg O , Ebbesson LOE , Nilsen TO , Jensen KH , Braithwaite VA . 2013 Environmental enrichment promotes neural plasticity and cognitive ability in fish. Proc. R. Soc. B **280** , 20131331. (10.1098/rspb.2013.1331) PMC3735255 23902903

[26] Zebunke M , Puppe B , Langbein J . 2013 Effects of cognitive enrichment on behavioural and physiological reactions of pigs. Physiol. Behav. **118** , 70–79. (10.1016/j.physbeh.2013.05.005) 23680428

[27] Calisi RM , Bentley GE . 2009 Lab and field experiments: are they the same animal? Horm. Behav. **56** , 1–10. (10.1016/j.yhbeh.2009.02.010) 19281813

[28] Dehnel A . 1949 Studies on the genus Sorex L. Ann. Univ. Mariae Curie Skłodowska C **4** , 17–102.

[29] Lázaro J , Dechmann DKN . 2021 Dehnel’s phenomenon. Curr. Biol. **31** , R463–R465. (10.1016/j.cub.2021.04.006) 34033763

[30] Pucek Z . 1965 Seasonal and age changes in the weight of internal organs of shrews. Acta Theriol. **10** , 369–438.

[31] Lázaro J , Hertel M , Sherwood CC , Muturi M , Dechmann DKN . 2018 Profound seasonal changes in brain size and architecture in the common shrew. Brain Struct. Funct. **223** , 2823–2840. (10.1007/s00429-018-1666-5) 29663134 PMC5995987

[32] LaPoint S , Keicher L , Wikelski M , Zub K , Dechmann DKN . 2016 Growth overshoot and seasonal size changes in the skulls of two weasel species. Dryad Digital Repository. (10.5061/DRYAD.G57G1) PMC5319358 28280592

[33] Nováková L , Lázaro J , Muturi M , Dullin C , Dechmann DKN . 2022 Winter conditions, not resource availability alone, may drive reversible seasonal skull size changes in moles. R. Soc. Open Sci. **9** , 220652. (10.1098/rsos.220652) 36133148 PMC9449468

[34] Lázaro J , Hertel M , LaPoint S , Wikelski M , Stiehler M , Dechmann DKN . 2017 Cognitive skills of common shrews (Sorex araneus) vary with seasonal changes in skull size and brain mass. J. Exp. Biol. **221** , 166595. (10.1242/jeb.166595) 29170257

[35] Lázaro J , Hertel M , Muturi M , Dechmann DKN . 2019 Seasonal reversible size changes in the braincase and mass of common shrews are flexibly modified by environmental conditions. Sci. Rep. **9** , 2489. (10.1038/s41598-019-38884-1) 30792434 PMC6385354

[36] Pucek Z . 1963 Seasonal changes in the braincase of some representatives of the genus Sorex from the palearctic. J. Mammal. **44** , 523–536. (10.2307/1377135)

[37] Yaskin VA . 1984 Seasonal changes in brain morphology in small mammals. Winter Ecol. Small Mamm. **10** , 183–191.

[38] Schaeffer PJ , O’Mara MT , Breiholz J , Keicher L , Lázaro J , Muturi M , Dechmann DKN . 2020 Metabolic rate in common shrews is unaffected by temperature, leading to lower energetic costs through seasonal size reduction. R. Soc. Open Sci. **7** , 191989. (10.1098/rsos.191989) 32431881 PMC7211839

[39] Churchfield S . 1990 The natural history of shrews. London, UK: A. & C. Black.

[40] Berthold P , Querner U . 1988 Was Zugunruhe wirklich ist—eine quantitative Bestimmung mit Hilfe von Video-aufnahmen bei Infrarotlichtbeleuchtung. J. Ornithol. **129** , 372–375. (10.1007/BF01643380)

[41] Deutscher Wetterdienst . 2024 DWD, Deutscher Wetterdienst. Wetter un Klima aus einer Hand. See https://www.dwd.de/.

[42] Searle JB . 1984 Breeding the common shrew (Sorex araneus) in captivity. Lab. Anim. **18** , 359–363. (10.1258/002367784780865360) 6513501

[43] Rychlik L , Jancewicz E . 2002 Prey size, prey nutrition, and food handling by shrews of different body sizes. Behav. Ecol. **13** , 216–223. (10.1093/beheco/13.2.216)

[44] Ashburner J , Friston KJ . 2000 Voxel-based morphometry—the methods. NeuroImage **11** , 805–821. (10.1006/nimg.2000.0582) 10860804

[45] Baldoni C , Thomas WR , von Elverfeldt D , Reisert M , Làzaro J , Muturi M , Dávalos LM , Nieland JD , Dechmann DKN . 2023 Histological and MRI brain atlas of the common shrew, Sorex araneus, with brain region-specific gene expression profiles. Front. Neuroanat. **17** , 1168523. (10.3389/fnana.2023.1168523) 37206998 PMC10188933

[46] Avants BB , Tustison NJ , Song G , Cook PA , Klein A , Gee JC . 2011 A reproducible evaluation of ANTs similarity metric performance in brain image registration. Neuroimage **54** , 2033–2044. (10.1016/j.neuroimage.2010.09.025) 20851191 PMC3065962

[47] Mazza V , Eccard JA , Zaccaroni M , Jacob J , Dammhahn M . 2018 The fast and the flexible: cognitive style drives individual variation in cognition in a small mammal. Anim. Behav. **137** , 119–132. (10.1016/j.anbehav.2018.01.011)

[48] Jones CM , Papanicolaou A , Mironidis GK , Vontas J , Yang Y , Lim KS , Oakeshott JG , Bass C , Chapman JW . 2015 Genomewide transcriptional signatures of migratory flight activity in a globally invasive insect pest. Mol. Ecol. **24** , 4901–4911. (10.1111/mec.13362) 26331997 PMC5102652

[49] Vollmayr B , Henn FA . 2003 Stress models of depression. Clin. Neurosci. Res. **3** , 4. (10.1016/s1566-2772(03)00086-0) 15925700

[50] Webber ES , Chambers NE , Kostek JA , Mankin DE , Cromwell HC . 2015 Relative reward effects on operant behavior: incentive contrast, induction and variety effects. Behav. Process. **116** , 87–99. (10.1016/j.beproc.2015.05.003) PMC4458168 25979604

[51] Bouton ME , Todd TP , Miles OW , León SP , Epstein LH . 2013 Within- and between-session variety effects in a food-seeking habituation paradigm. Appetite **66** , 10–19. (10.1016/j.appet.2013.01.025) 23434973 PMC3646953

[52] Mallory CS , Hardcastle K , Campbell MG , Attinger A , Low IIC , Raymond JL , Giocomo LM . 2021 Mouse entorhinal cortex encodes a diverse repertoire of self-motion signals. Nat. Commun. **12** , 671. (10.1038/s41467-021-20936-8) 33510164 PMC7844029

[53] Strekalova T , Spanagel R , Dolgov O , Bartsch D . 2005 Stress-induced hyperlocomotion as a confounding factor in anxiety and depression models in mice. Behav. Pharmacol. **16** , 171–180. (10.1097/00008877-200505000-00006) 15864072

[54] Ochocińska D , Taylor JRE . 2005 Living at the physiological limits: field and maximum metabolic rates of the common shrew (Sorex araneus). Physiol. Biochem. Zool. **78** , 808–818. (10.1086/431190) 16096983

[55] Keicher L , O’Mara MT , Voigt CC , Dechmann DKN . 2017 Stable carbon isotopes in breath reveal fast metabolic incorporation rates and seasonally variable but rapid fat turnover in the common shrew (Sorex araneus). J. Exp. Biol. **220** , 2834–2841. (10.1242/jeb.159947) 28546508

[56] Levy-Gigi E , Szabó C , Kelemen O , Kéri S . 2013 Association among clinical response, hippocampal volume, and FKBP5 gene expression in individuals with posttraumatic stress disorder receiving cognitive behavioral therapy. Biol. Psychiatry **74** , 793–800. (10.1016/j.biopsych.2013.05.017) 23856297

[57] Zannas AS , Wiechmann T , Gassen NC , Binder EB . 2016 Gene–stress–epigenetic regulation of FKBP5: clinical and translational implications. Neuropsychopharmacology **41** , 261–274. (10.1038/npp.2015.235) 26250598 PMC4677131

[58] McNamara JM , Buchanan KL . 2005 Stress, resource allocation, and mortality. Behav. Ecol. **16** , 1008–1017. (10.1093/beheco/ari087)

[59] Hamet P , Tremblay J . 2005 Genetics and genomics of depression. Metabolism **54** , 10–15. (10.1016/j.metabol.2005.01.006) 15877306

[60] Lu Q *et al* . 2019 Chronic unpredictable mild stress-induced behavioral changes are coupled with dopaminergic hyperfunction and serotonergic hypofunction in mouse models of depression. Behav. Brain Res. **372** , 112053. (10.1016/j.bbr.2019.112053) 31288060

[61] Wang X , Xu J , Wang Q , Ding D , Wu L , Li Y , Wu C , Meng H . 2021 Chronic stress induced depressive-like behaviors in a classical murine model of Parkinson’s disease. Behav. Brain Res. **399** , 112816. (10.1016/j.bbr.2020.112816) 32783904

[62] Baldoni C . 2024 Data and code for ‘Captivity alters behavior but not seasonal brain size change in semi-naturally housed shrews’. Edmond. (10.17617/3.XMQBQH) PMC12105801 40420851

[63] Baldoni C , Raptis K , Farantouri M , Lenzi I , Lim J , Menz M *et al* . 2025. Supplementary Material from: Captivity Alters Behavior but Not Seasonal Brain Size Change in Semi–Naturally Housed Shrews. FigShare (10.6084/m9.figshare.c.7667237) PMC12105801 40420851

